# Supplementary material for: Impact of micro-habitat fragmentation on microbial population growth dynamics
Source: ISME J. 2024 Dec 23;19(1):wrae256. doi: 10.1093/ismejo/wrae256 (PMC11964898; doi:10.1093/ismejo/wrae256)
Supplement: Supplementary_ISME_final_251124_revised_171224 [file supplementary_isme_final_251124_revised_171224.pdf]

Supplementary Information

Impact of micro-habitat fragmentation on microbial population growth dynamics

Dina Mant*, Tomer Orevi*, Nadav Kashtan

SI Includes:

1. Supporting Figures

2. Supporting Tables

Supplementary Figures

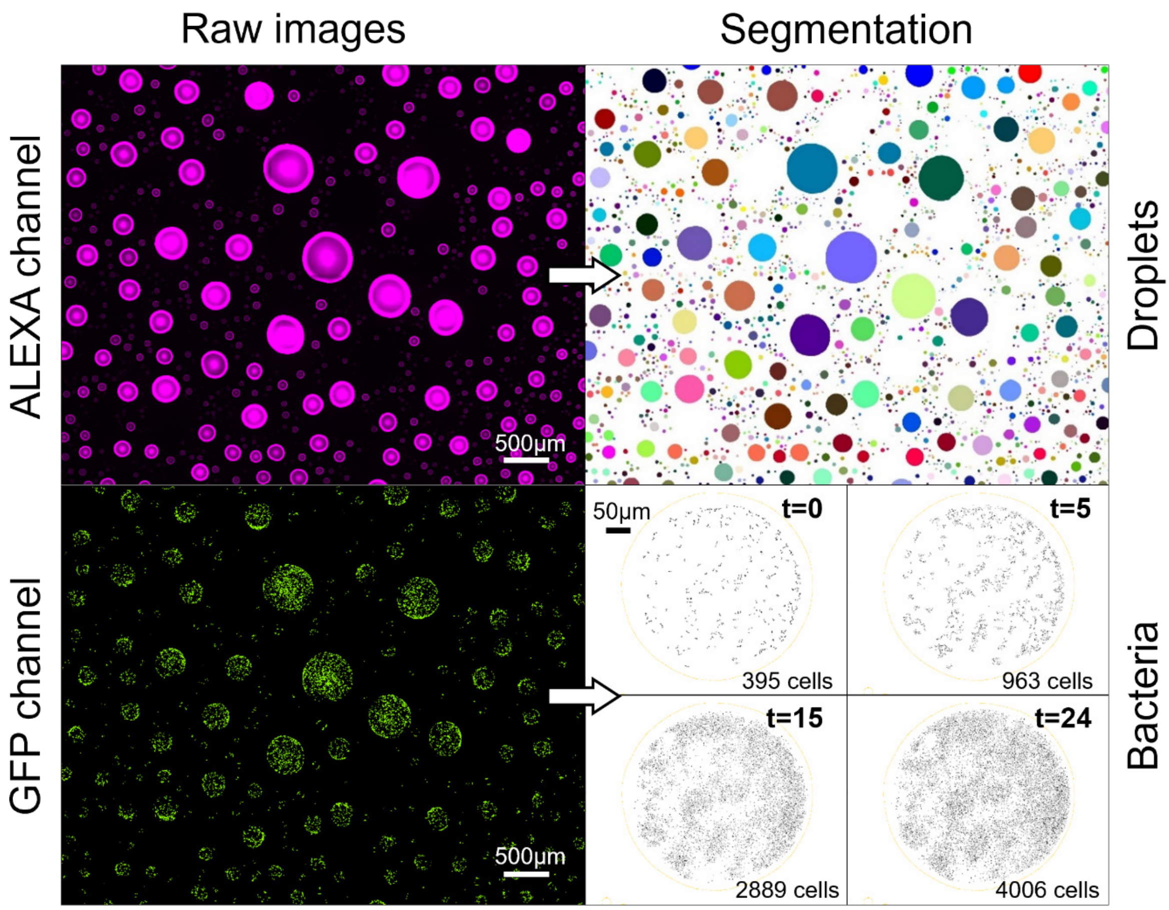

Figure S1. **Image processing results. Left:** two channels raw data image of a section of a single μ-SPLASH chip. Alexa 647 channel captures the droplet boundaries. GFP channel captures bacterial cells. **Right:** droplets (top) and bacterial cell (bottom) segmentations. Bacterial cell counts based on segmentation (in a single droplet) shown at different time points.

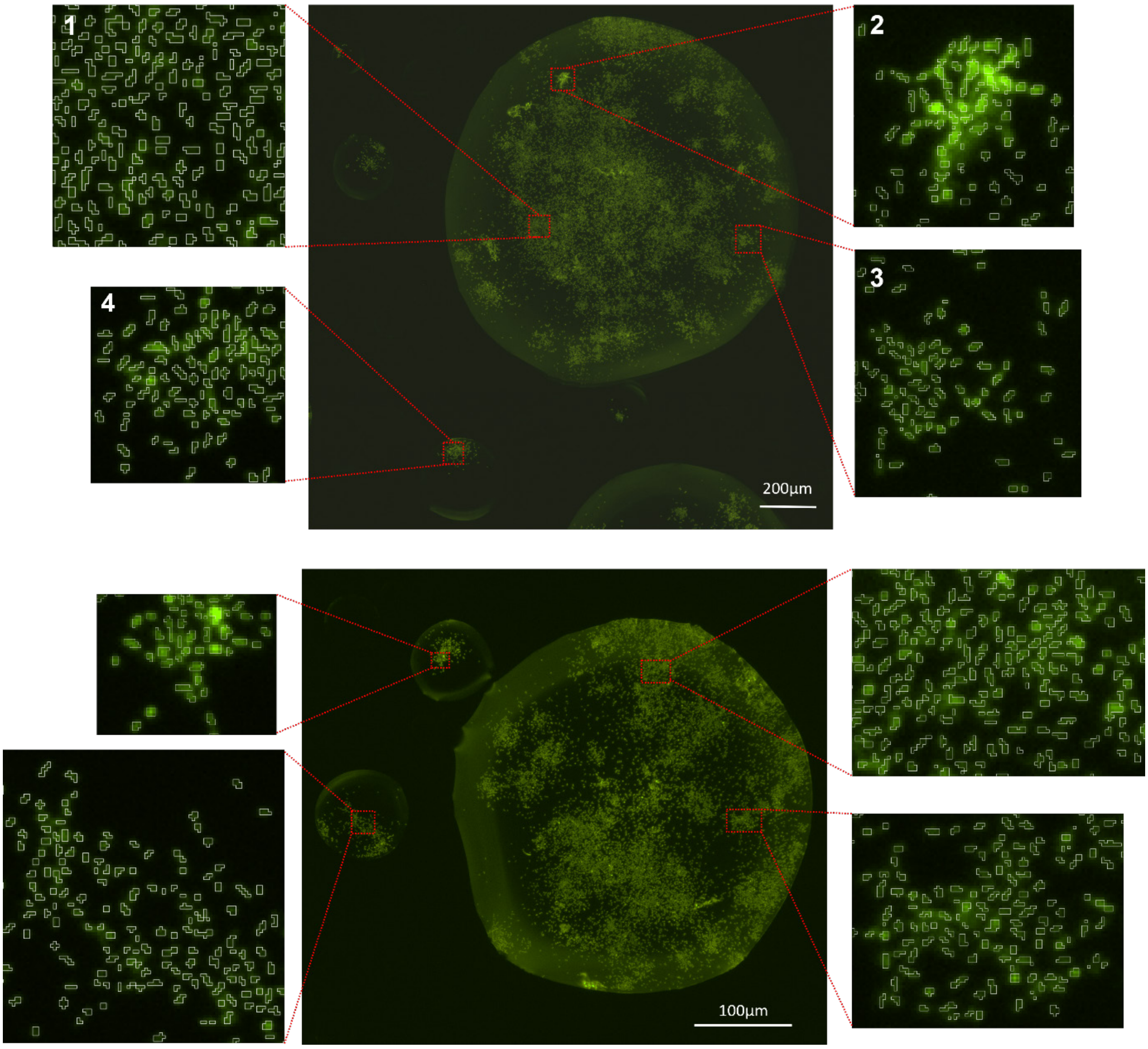

Figure S2. Single-cell segmentation within representative droplets of various sizes. Representative images of droplets of various size captured at t=24 h. Each white-bordered polygon marks a single bacterial cell as identified by the segmentation algorithm. The upper image represents the larger volume size range (volume = $10^{6.7}$ µm^3) and contains 15,042 bacterial cells. As can be seen, cells are not evenly distributed within the droplet and are mostly organized as a mono-layer of surface-attached cells. Some areas are less densely populated (inset 1,3) and some more densely populated (inset 2), sometime forming small aggregates. The less densely populated areas are common and cell segmentation therein is typically accurate. The more densely populated areas are less common and segmentation therein occasionally exhibit some degree of underestimation of cell numbers. The smaller droplet in the upper panel is of intermediate size (volume = $10^{4.4}$ µm^3) and contains 139 bacterial cells, which are concentrated in one, relatively densely populated area (inset 4), demonstrating that such aggregation can occur not only in large droplets. Even so, the segmentation in the densely populated area within the intermediate-sized droplet does not lead to any significant underestimation of bacterial cells. The lower panel shows examples of single-cell segmentation within several other small and large droplets.

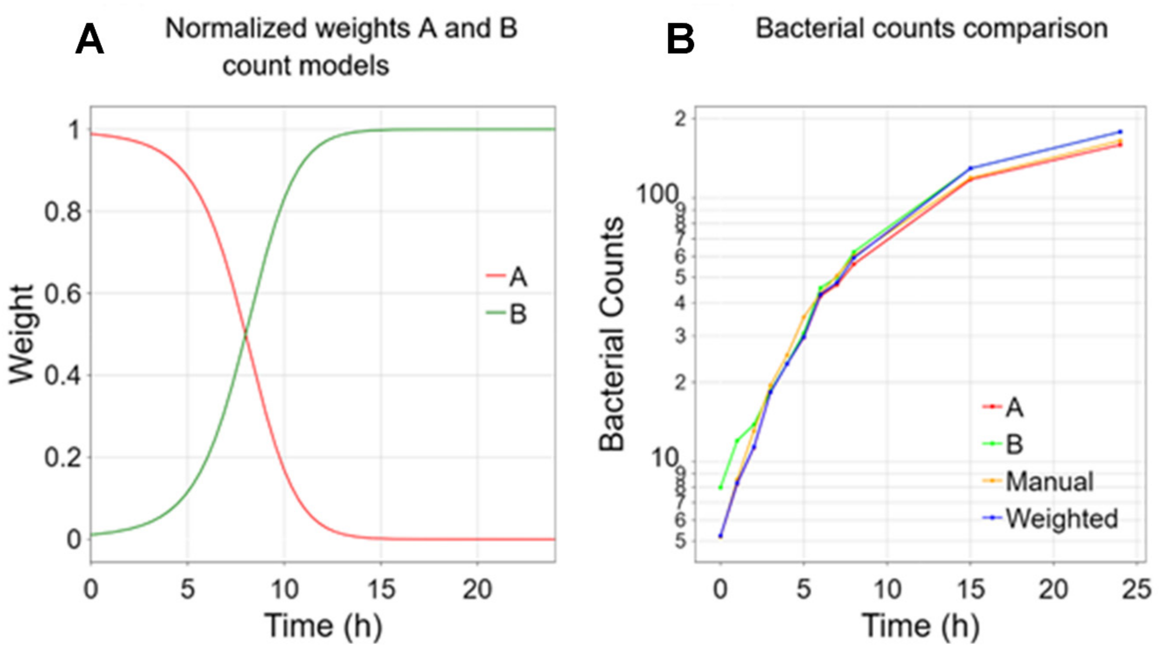

Figure S3. Combining two trained-models for bacterial segmentation for the estimation of cell counts. (A) The normalized weights of bacterial segmentation counting models A and B (early and late hours, correspondingly) throughout the experiment duration (0-24 hours). Model A was trained on hours 0-7 and therefore has higher relative weight throughout this time frame, and *vice versa* with model B. **(B)** Comparison of the different bacterial counting methods in droplets: segmentation models A and B, manual counting, and the weighted segmentation (in red, green, orange and blue, respectively), throughout the experiment duration. Comparable cell counts were achieved by the models and manual cell counts of 54 representative droplets.

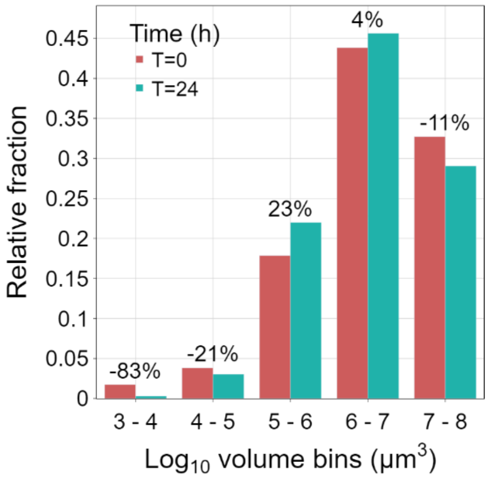

Figure S4. Relative change of bacterial population size at different droplet size bins. The relative change (in %) of bacterial population fraction for different droplet size $\log_{10}$ bins between the initial and final time points (t=0 h; red bars and t=24 h; green bars). Data taken from experiment chip C6HD. Positive values denote a relative increase in the fraction of the metapopulation residing within that bin whereas negative values indicate a decrease.

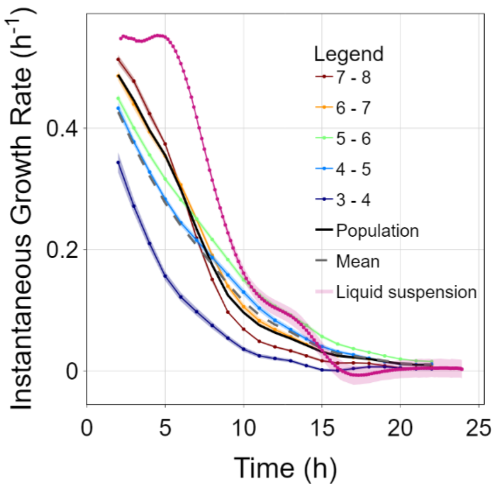

Figure S5. Instantaneous growth rates over time. The instantaneous growth rate of each volume bin through time. Data shown for chip C6HD. Instantaneous growth calculated from the slope of the curve plotting the natural log of the number of bacterial cells within each droplet over a moving time window of 4 hours. The instantaneous growth rates measured in liquid suspension (based on OD measurements) under the same medium and conditions is shown for comparison (pink line).

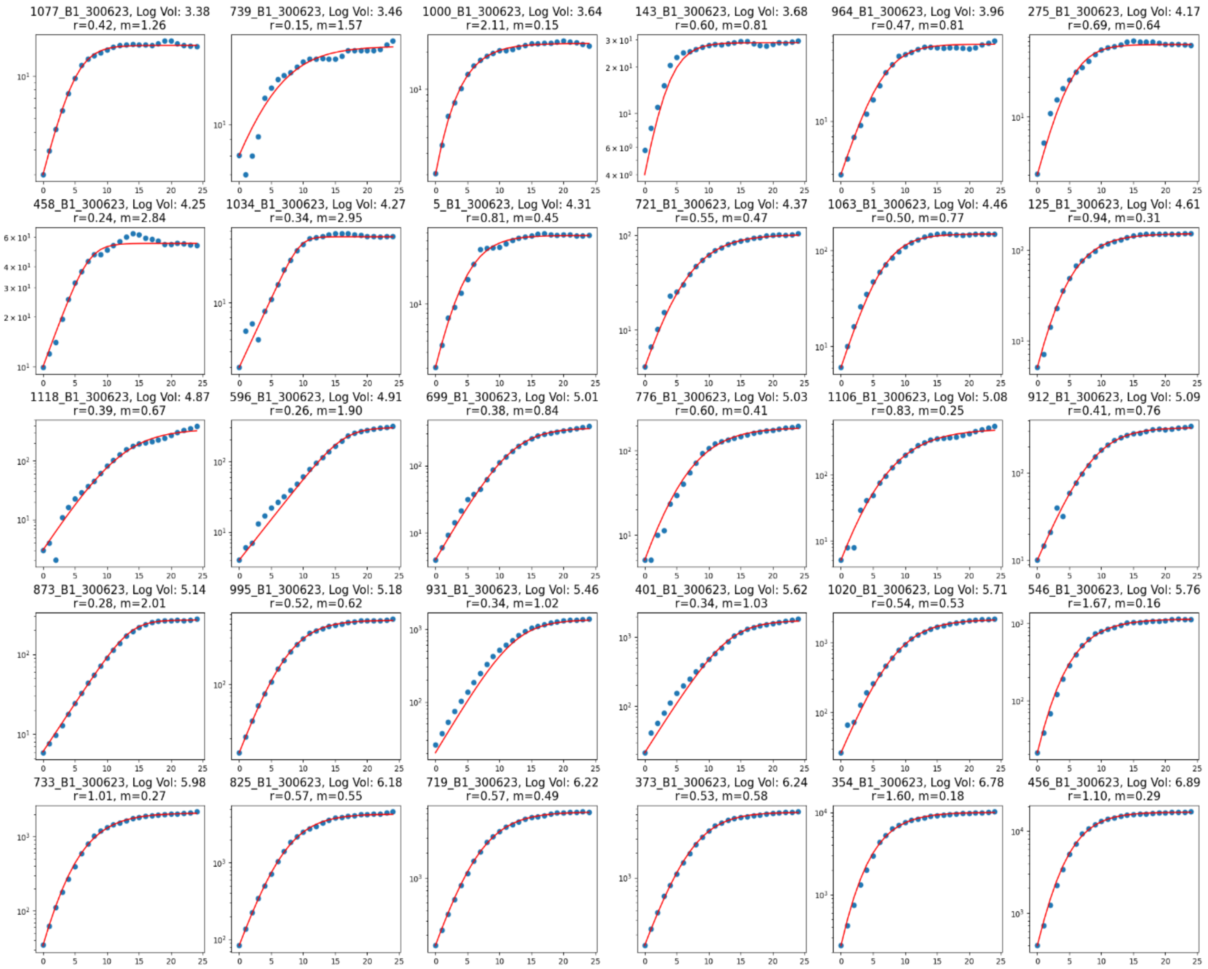

Figure S6. Representative sample of growth curves extracted from individual droplets and fitted to a generalized logistic growth model. The blue dots represent the experimental cell count (log-scale) for each time point. The red line represents the fitted model. The sampled subplots are ordered based on the droplets volume from left to right. The fitted model parameters r and m are presented above each plot.

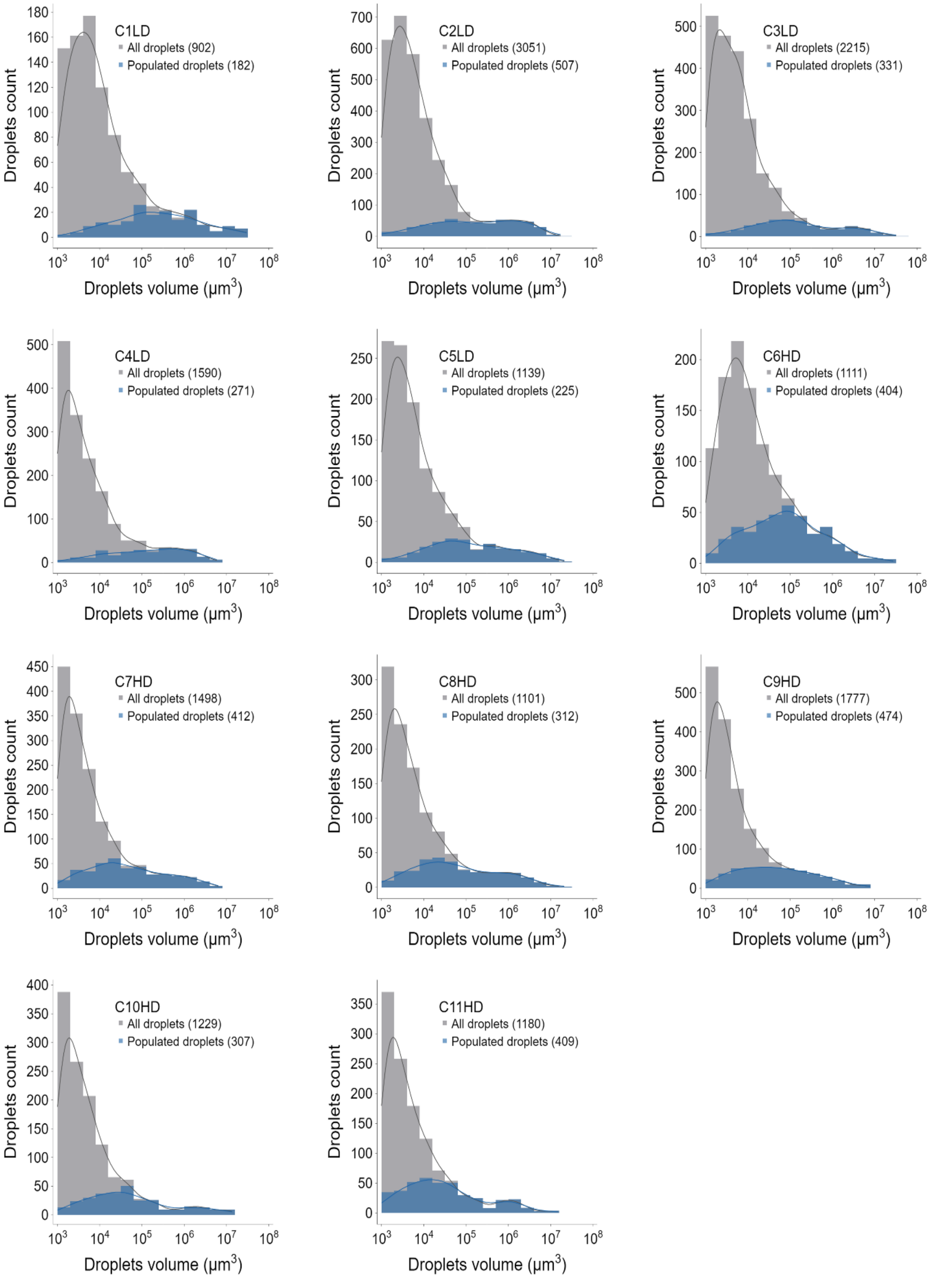

Figure S7. Droplets' volume distribution across 11 chips (C1-C11; X axis in log scale). Gray bars denote all droplets while blue bars denote bacteria populated droplets.

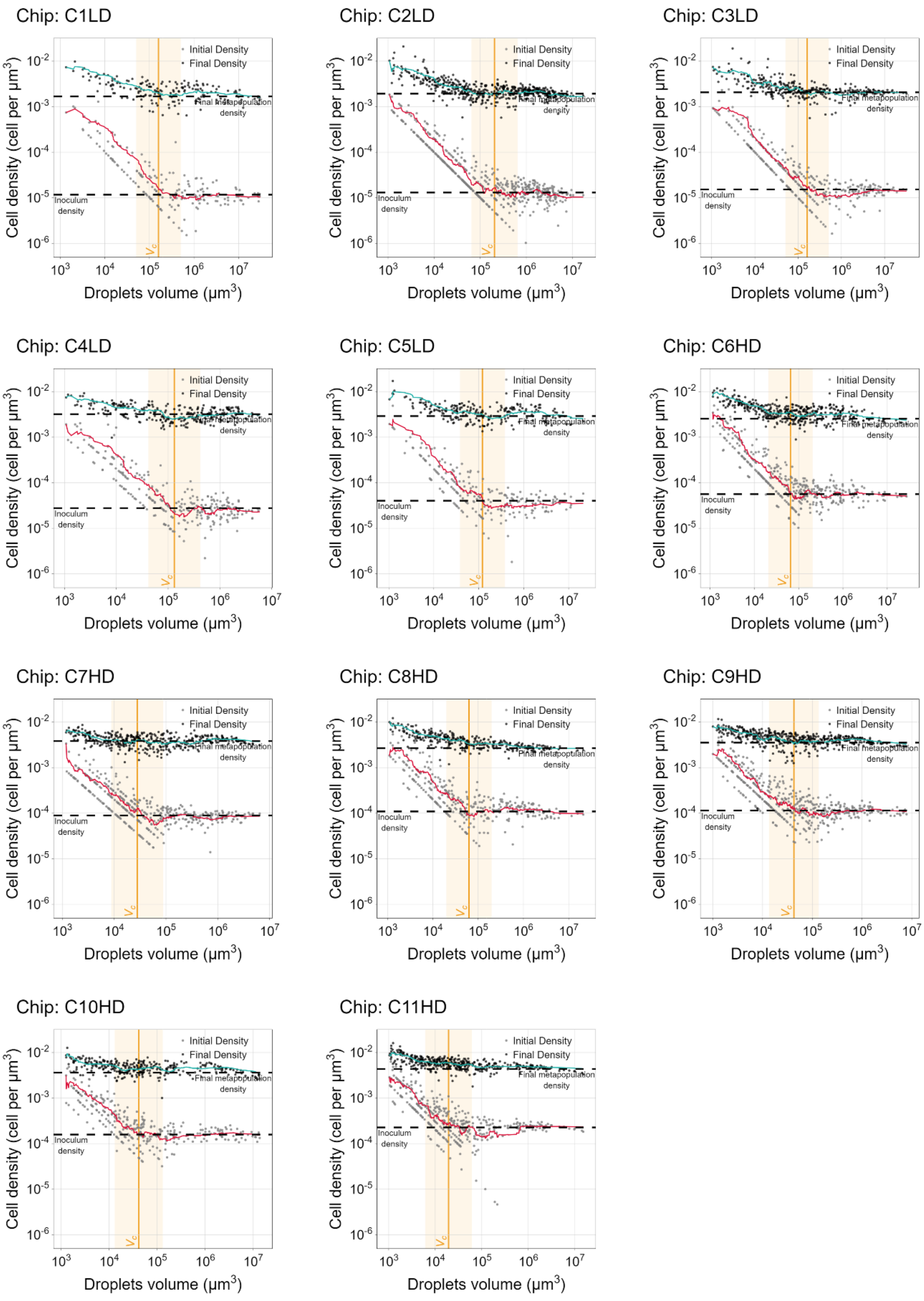

Figure S8. Initial and final cell density at t=0 h and t=24 h across 11 chips. The red and cyan line denotes a moving average of log-transformed values (window=25). The black dashed line represents the metapopulation initial and final cell density. The vertical orange line represents the critical droplet volume (Vc) of each chip. The background orange shade represents the intermediate droplets range.

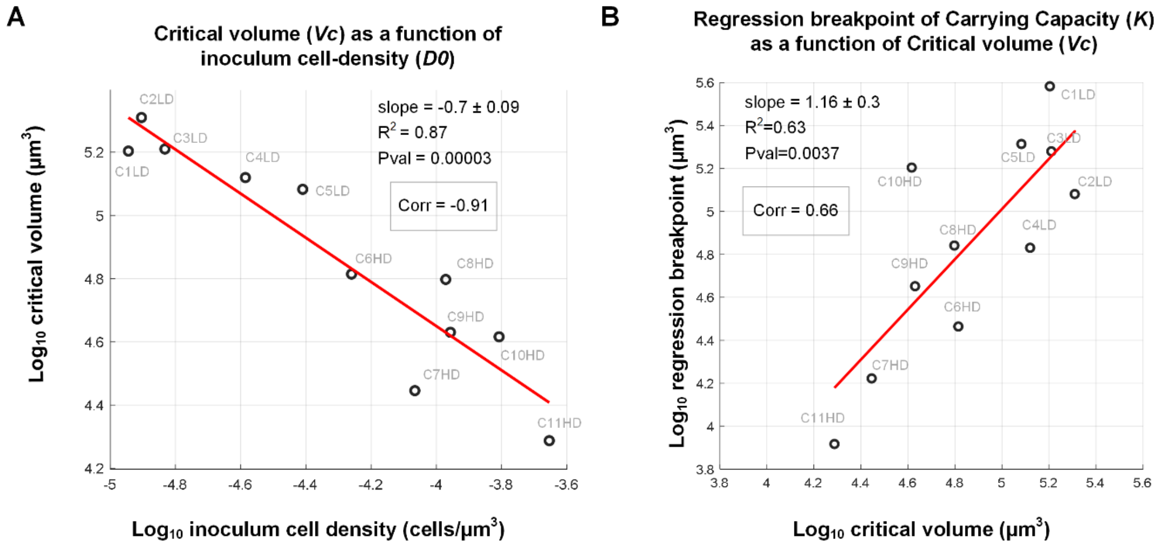

Figure S9. Critical volume (*Vc*) across the 11 chips. (A) *Vc* is defined as the droplet volume at which the mean initial cell density converges to the inoculum cell density. *Vc* values were computed for each chip as the volume where a moving average (window size=100) of log10 cell density at time t = 0 h reaches 99% of the log10 inoculum density. *Vc* shows a strong negative correlation with the chip inoculum density, as expected (r = –0.91; spearman correlation). **(B)** The breakpoints of the regression between K and droplet volume were determined by minimizing the sum of squares errors for two-phase regression fitting across the entire droplet volume range. These breakpoints highly correlate with the *Vc* values across chips.

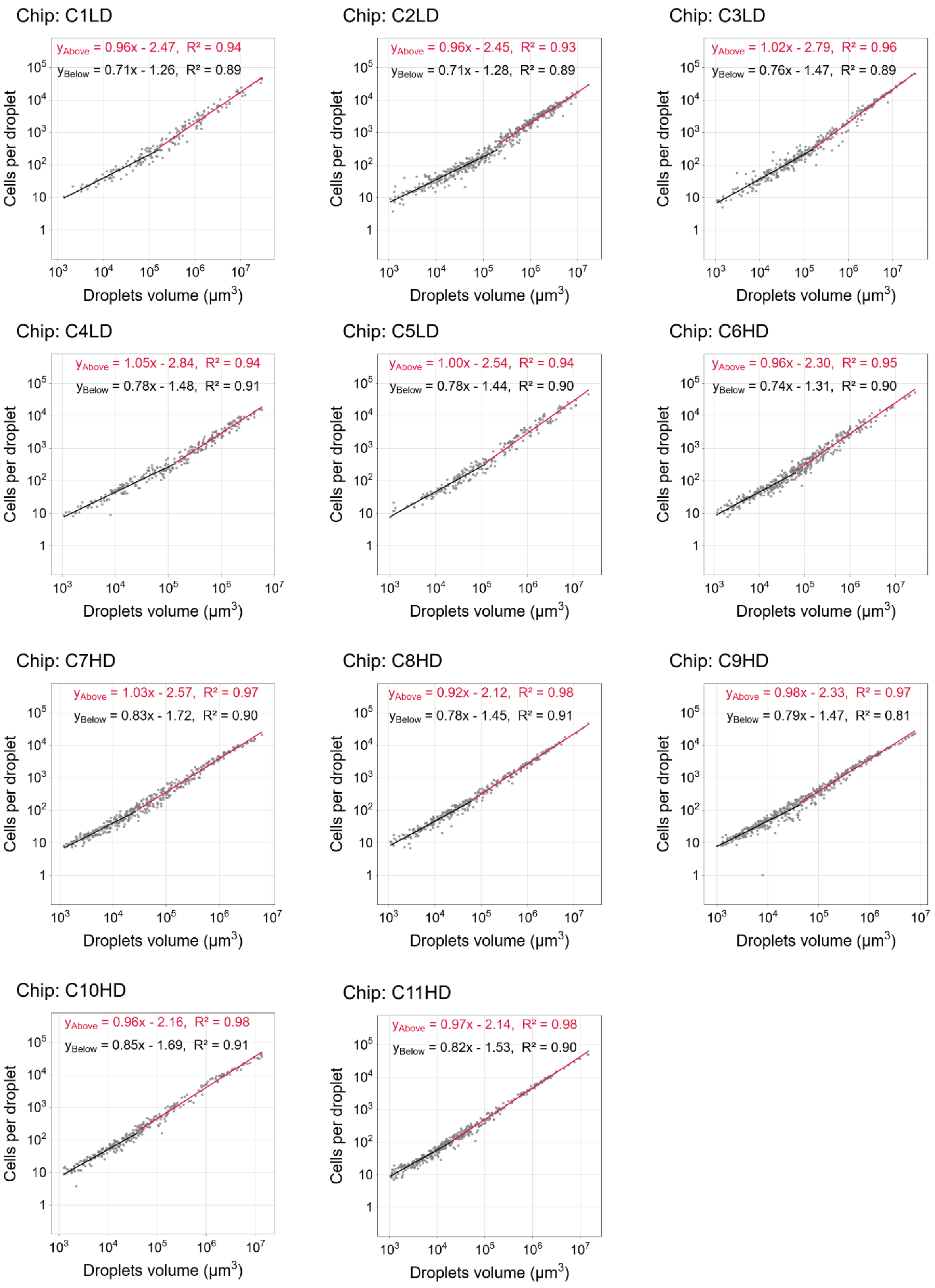

Figure S10. The relation between carrying capacity (*K*) and droplet volume (*V*) (log-log plot) across chips. *K* shows two sub-linear phases with different exponents for each chip. The exponents of these two phases are similar across chips.

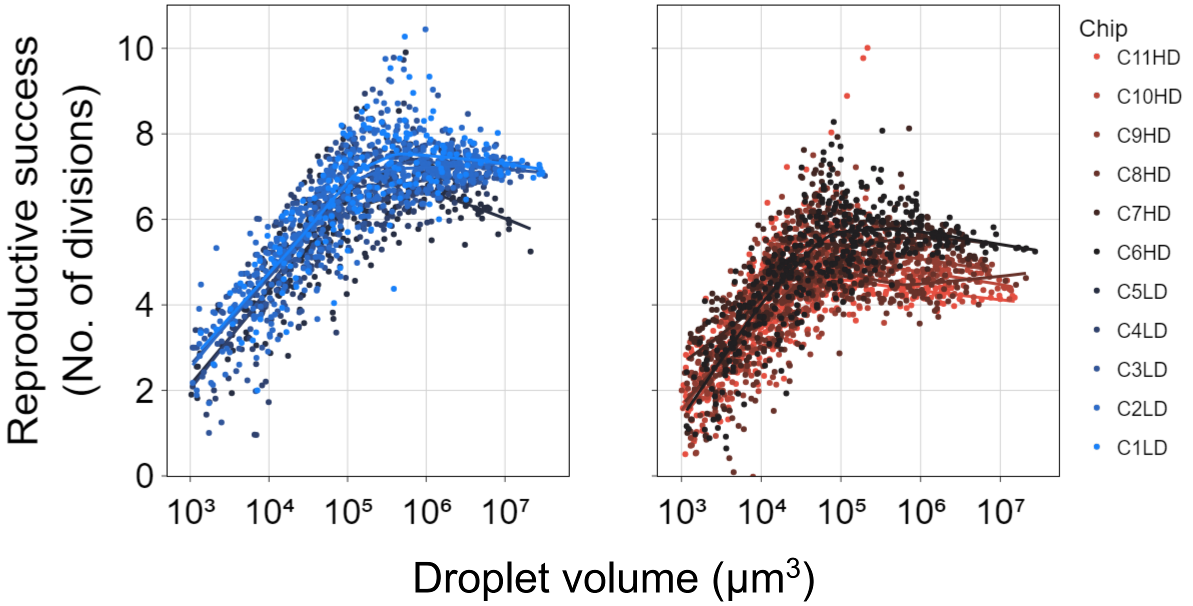

Figure S11. Reproductive success (*RS*) across 11 chips. Left: low density chips (LD; chip's 1-5) in blue. **Right:** high density chips (HD; chip's 6-11) in red. The colored lines represent the locally weighted scatterplot smoothing (LOWESS, frac=0.4) trendline of each respective chip.

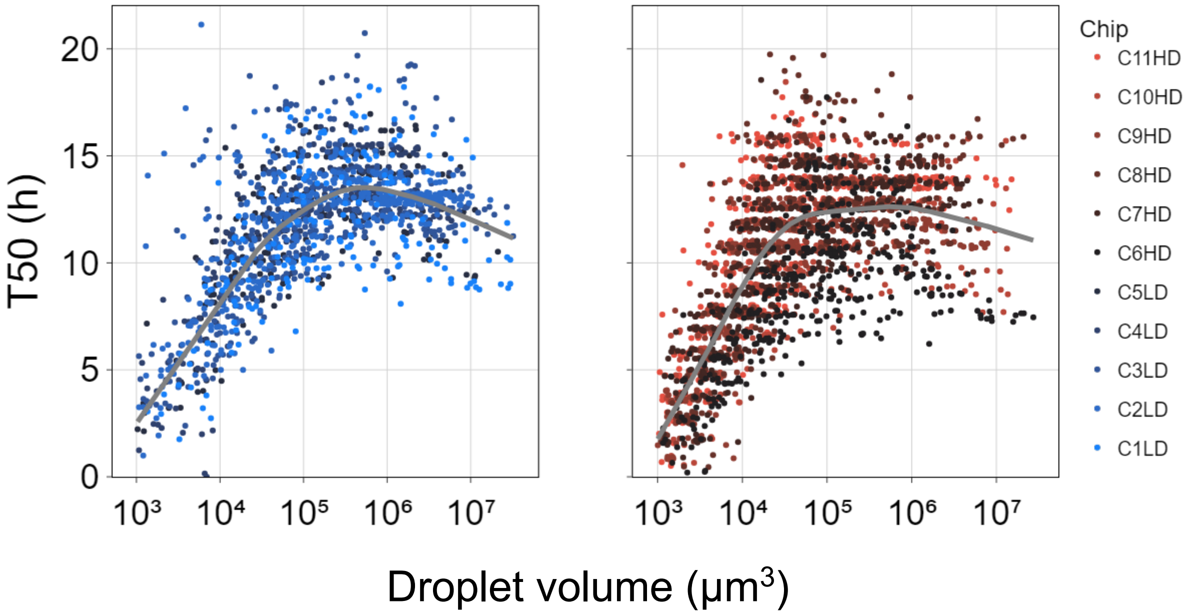

Figure S12. Time to reach 50% of the carrying capacity (T_{50}) across 11 chips. Left: low density chips (LD; chip's 1-5) in blue. **Right:** high density chips (HD; chip's 6-11) in red. The gray line represents the locally weighted scatterplot smoothing (LOWESS, frac=0.4) trendline of all the LD/HD chips respectively.

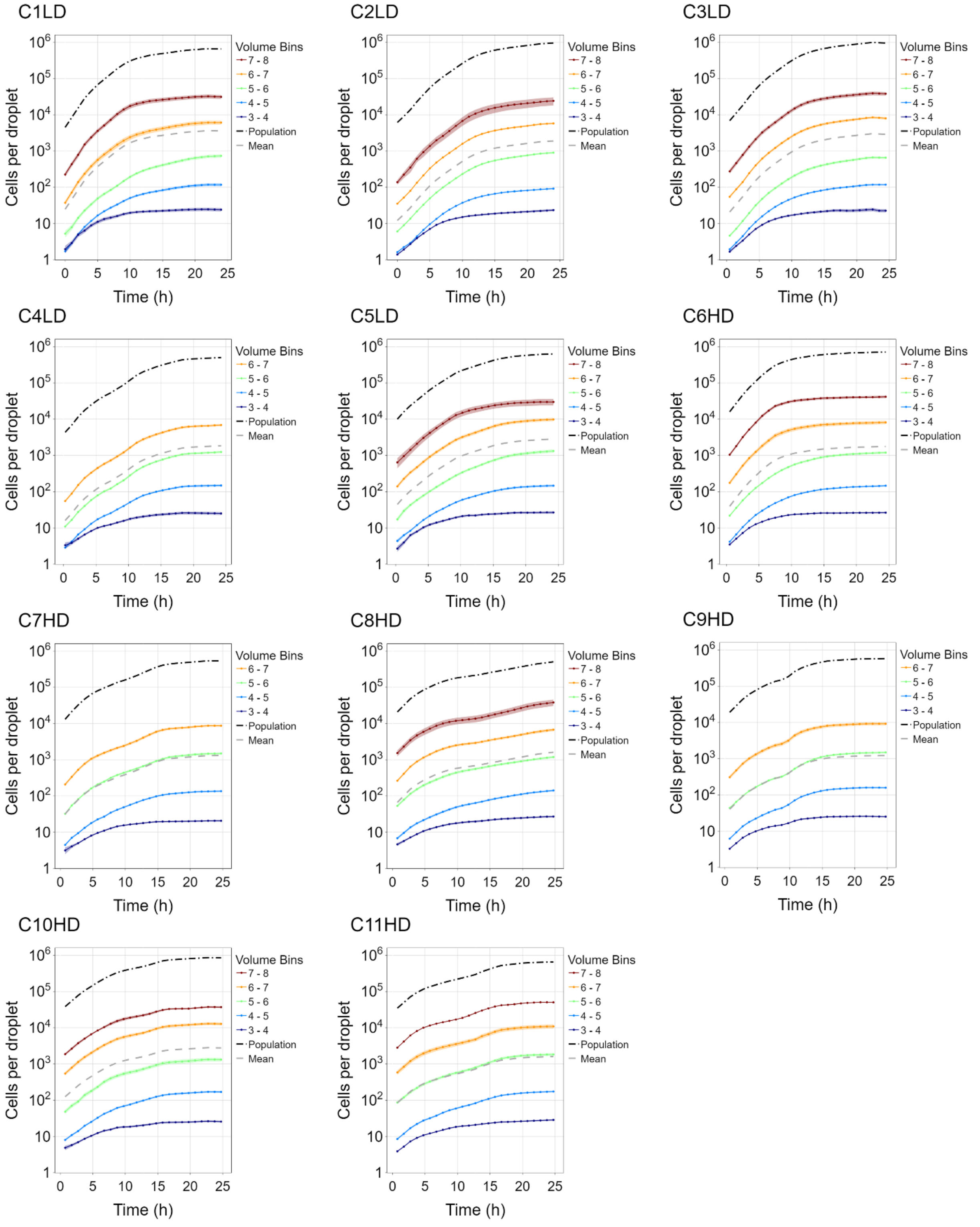

Figure S13. Mean growth curves across 11 chips. The colored lines represent the mean cell count per droplet bin volume ($\log_{10}$) over 24 hours. The shaded color of each curve represents the standard error.

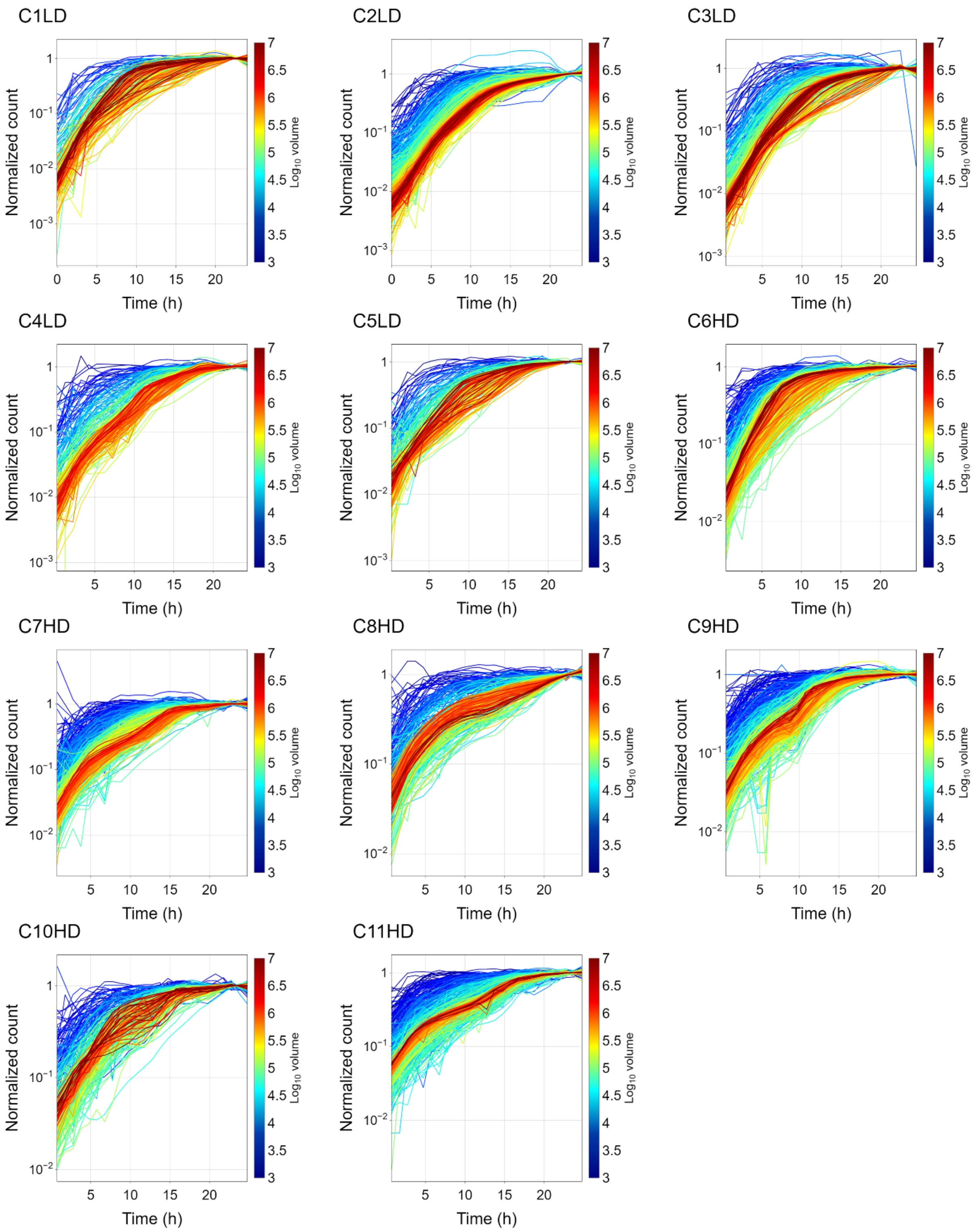

Figure S14. Normalized growth curves of individual droplets across all 11 chips. Normalized growth in each droplet represented by a line colored based on the droplet's volume. Y axis is in log scale.

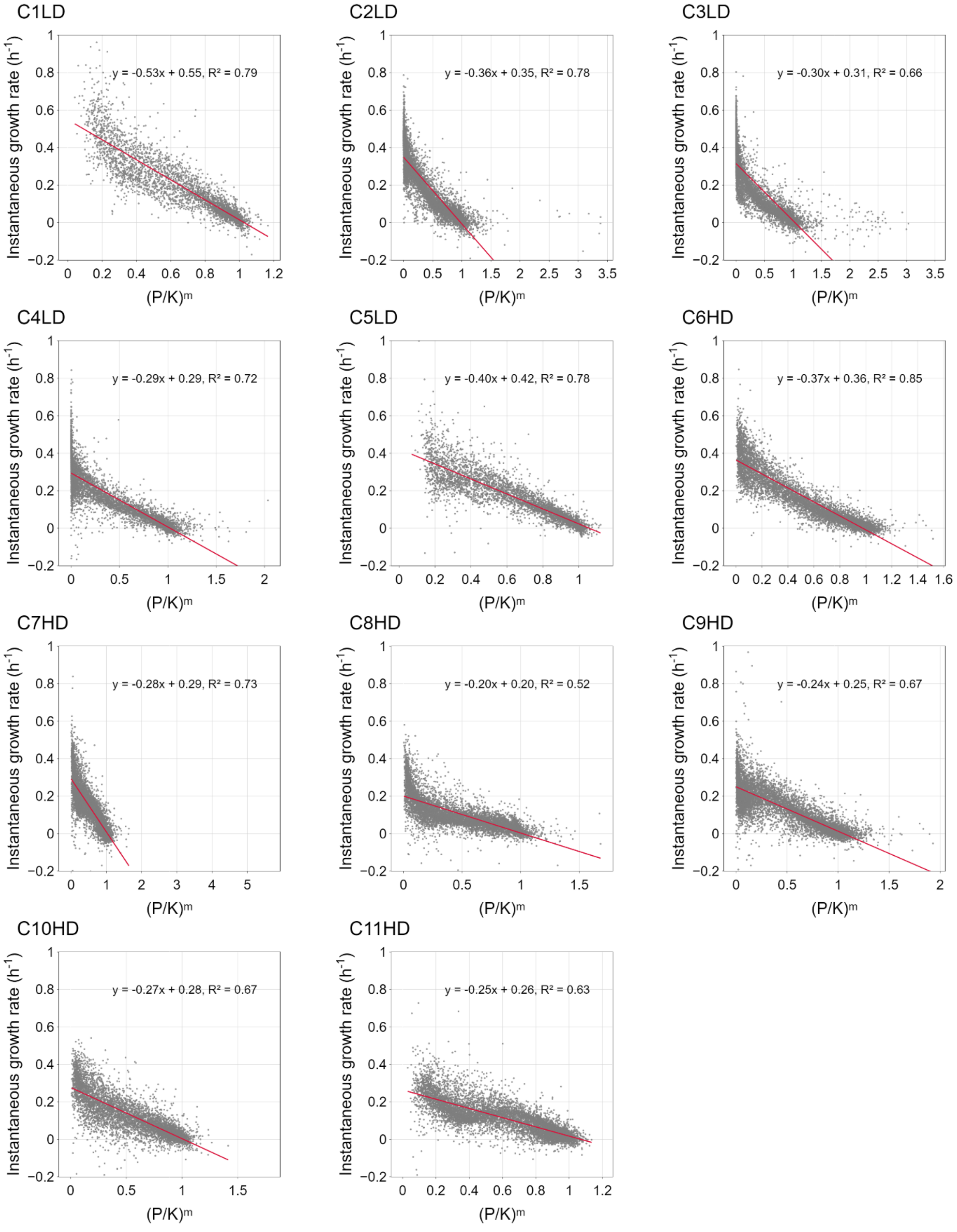

Figure S15. Instantaneous growth rates as a function of 'relative density' across 11 chips. The relative density is the ratio between cell number and carrying capacity in the power of 'm' (m is the fitted deceleration parameter). Mean deceleration parameter of each chip was calculated by averaging the deceleration parameters of all the droplets within that chip. Linear relationship represented by OLS regression line.

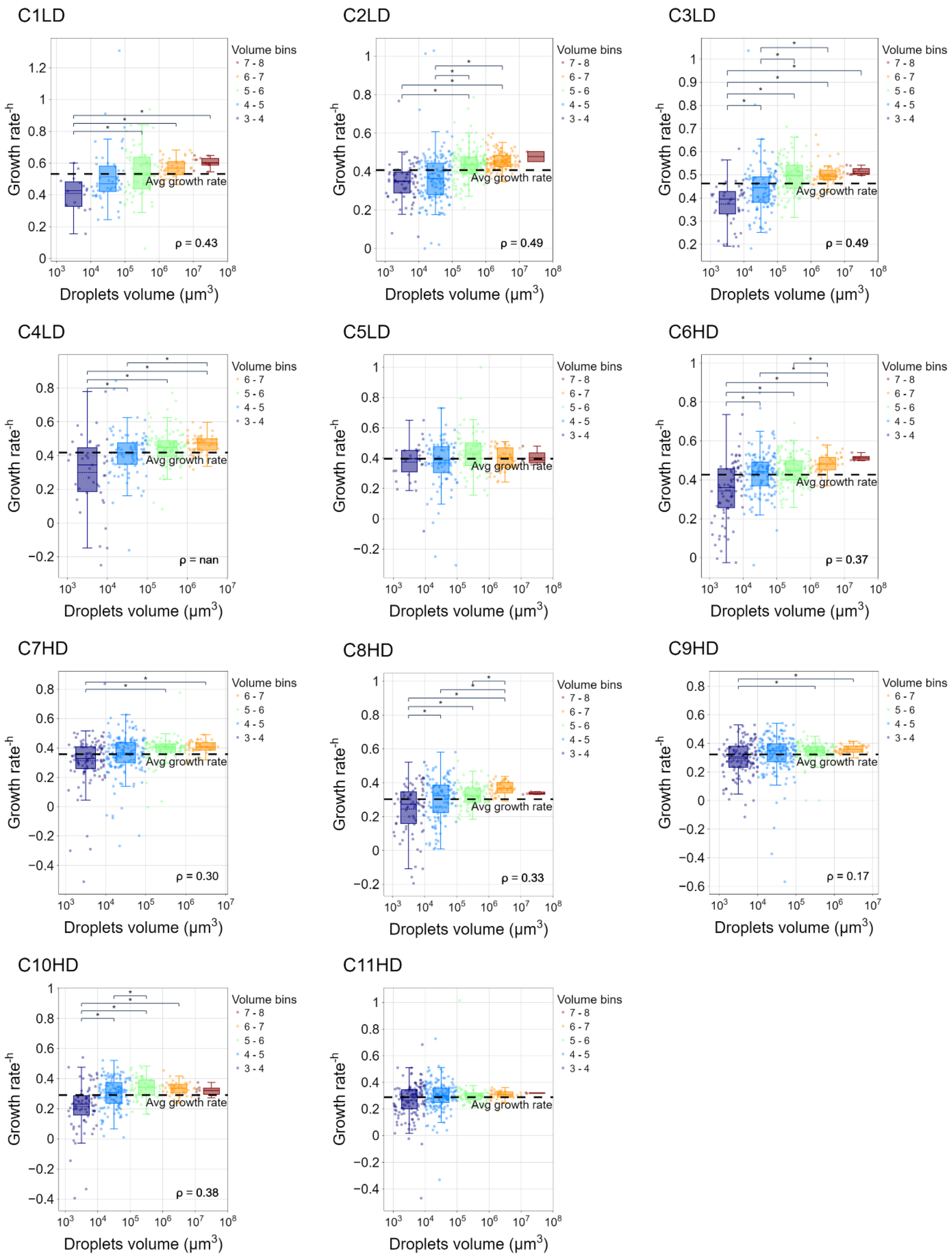

Figure S16. Maximal growth rates (μ_{max}) within individual droplets across all 11 chips. The boxplots represent the growth rate distribution of the droplets within each volume bin, colored based on the volume bins. The dashed line represents the overall metapopulation μ_{max} (of each chip). A permutation tests (n=1000) was used to test statistical significance between the bins' means (α=0.01).

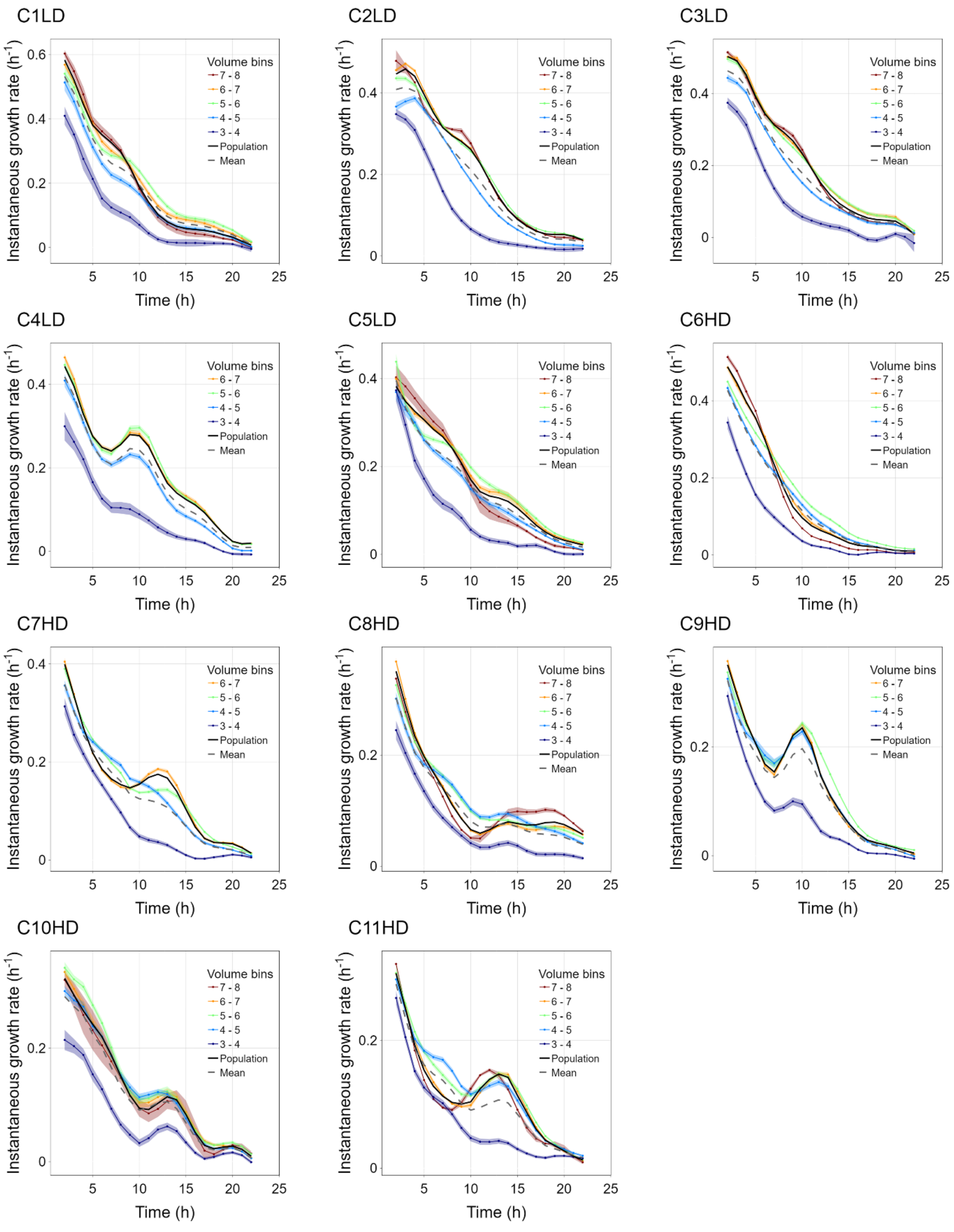

Figure S17. The instantaneous growth rate of each volume bin through time and across chips. Rates extracted from the slope of the curve plotting the natural log of the number of bacterial cells within each droplet over a moving time window of 4 hours. The shaded color of each line represents the standard error.

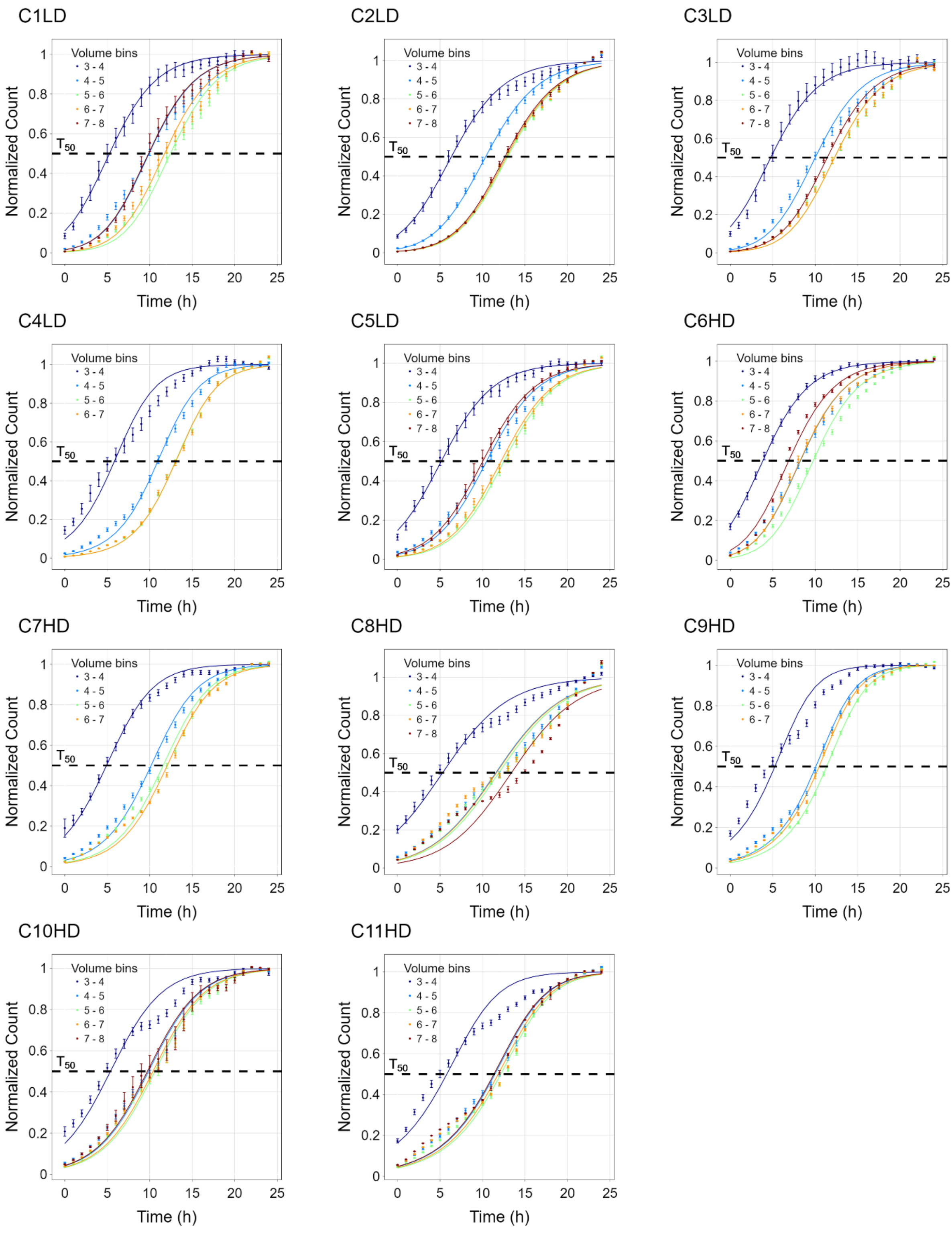

Figure S18. Normalized growth curves for each volume bin across chips. Dots and error bars represent Mean ± SE of the experimental data. The line represents the generalized logistic growth model for each volume bin, based on the median r and m values of all droplets, and the mean initial cell density.

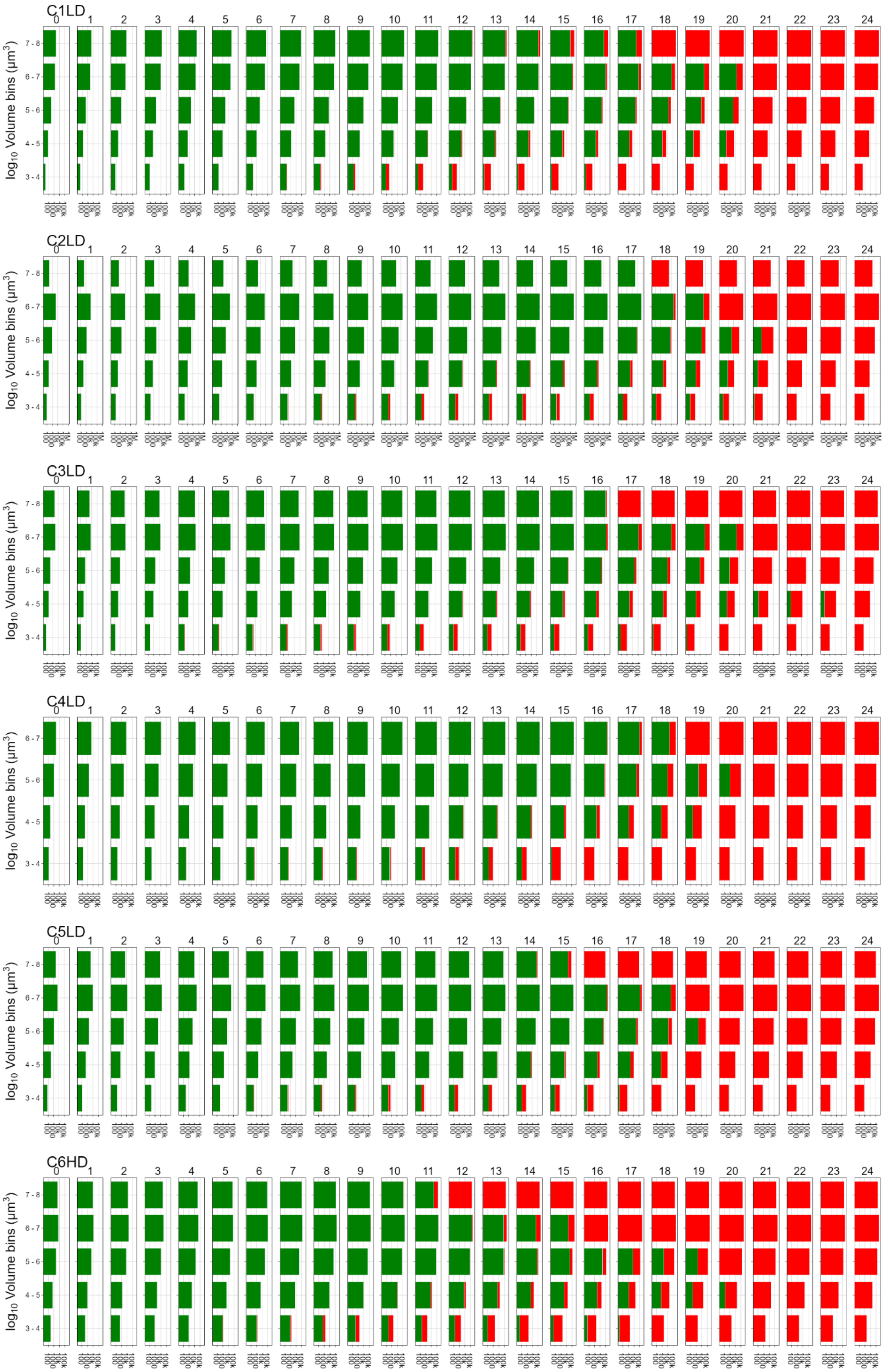
C1LD
C2LD
C3LD
C4LD
C5LD
C6HD
log10 Volume bins (µm3)
7-8
6-7
5-6
4-5
3-4

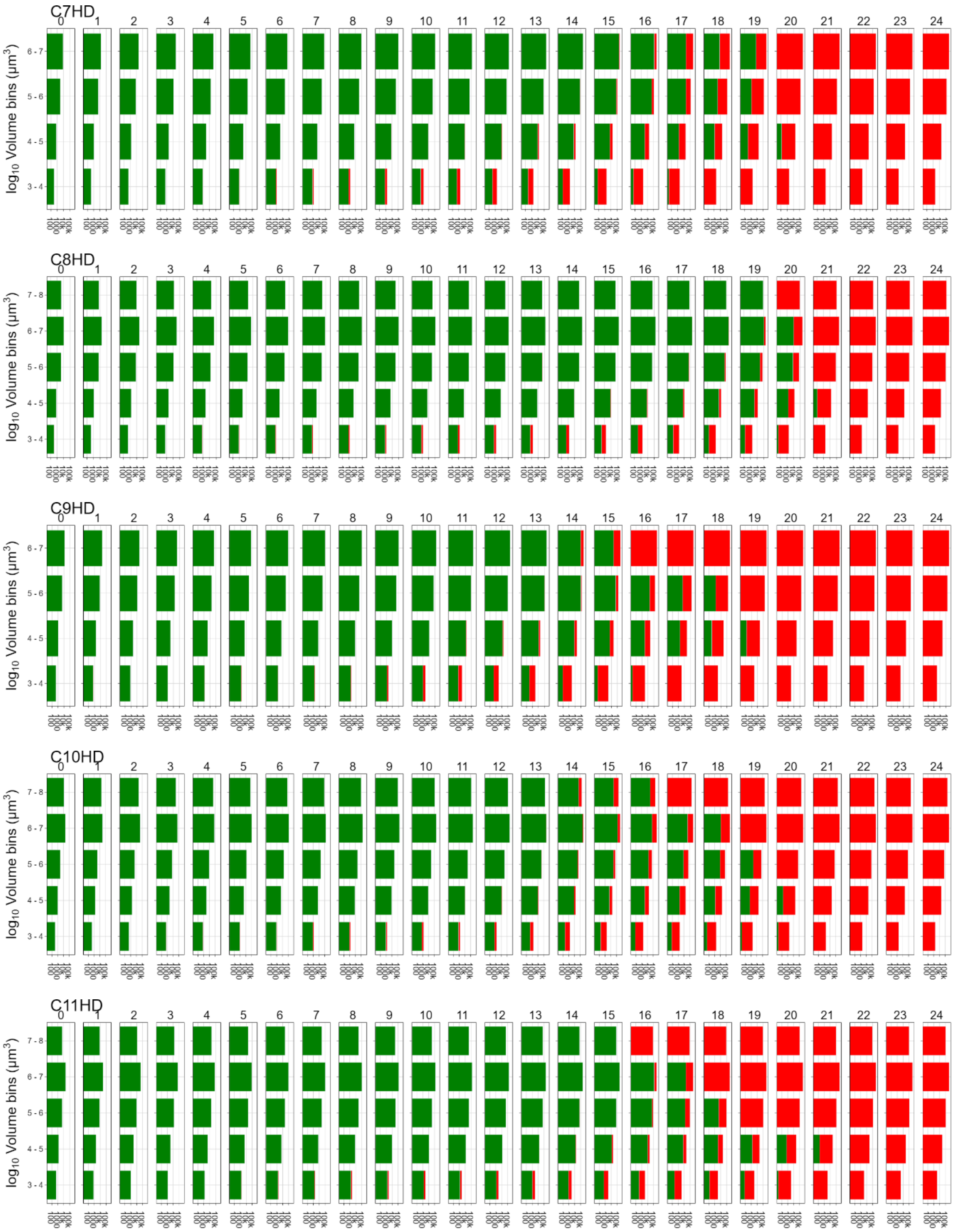

Figure S19. Bar chart representing time-lapse of the overall population dynamics and state (growing in GREEN; stationary in RED) over time in each bin, across chips. The bar width represents the cell counts (in log scale) in each drop-volume bin over time.

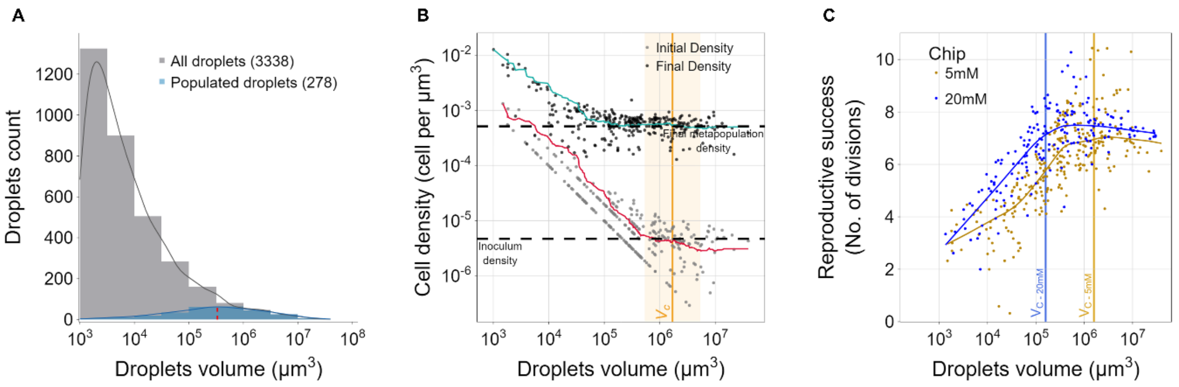

Figure S20. μ-SPLASH experiment with different nutrient concentrations. Analysis of a set of μ-SPLASH chips with lower nutrient concentrations (5 mM glucose and 10 mM tryptone compared to 10 mM glucose and 20 mM tryptone used in the 11 chips reported in this study). The results of these lower nutrients chips showed similar growth dynamics to the original experiment, yet with lower yields. The analysis was done on 278 droplets from six chips, combined. (A) The distribution of droplet volumes, were gray represent all the droplets and blue indicates droplets populated with bacteria. (B) Initial and final cell density at t=0 h and t=24h, correspondingly. The red and cyan lines denote a moving average of log-transformed values (window=25). The black dashed line represents the metapopulation initial and final cell density, i.e., the overall initial (or final) cell counts within all droplets divided by the total volume of all droplets. Note the lower inoculum cell density in comparison to the 11 chips C1-C11 analyzed in the main text. The vertical orange color represents the critical droplet volume (*Vc*), above which the mean initial cell density converged to the metapopulation cell density. The background orange shade represents the intermediate droplets range ($10^{5.7}$-$10^{6.7}$ μm^3). (C) Reproductive success as a function of droplet volume. The blue and orange dots represent the 10 mM glucose chip (C1LD) and the combined 5 mM glucose chips, respectively. The curves represent the locally weighted scatterplot smoothing (LOWESS, frac=0.5) trendline of each experiment. The respective vertical lines represent *Vc* of each experiment.

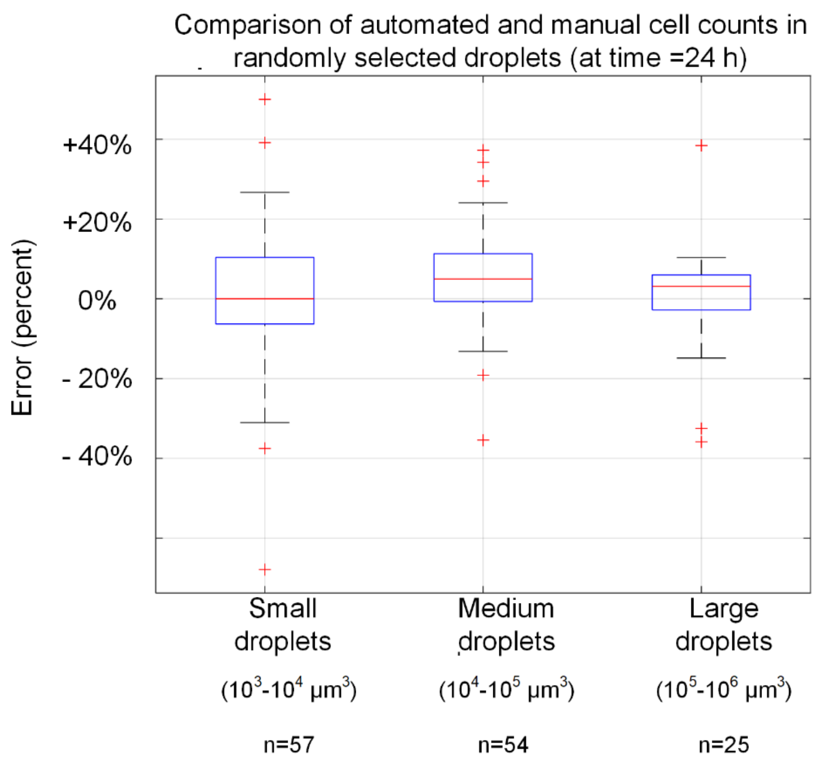

Figure S21. Estimation of cell number quantification error rates. Comparison of automated and manual cell counts in randomly selected droplets at t=24 h. Box plots represent the error (percent) for small, medium, and large droplets. The box plots display the median (red line), 25th and 75th percentiles (box edges), and outliers (red crosses). Sample sizes (n) for each droplet size category are shown below the x-axis.

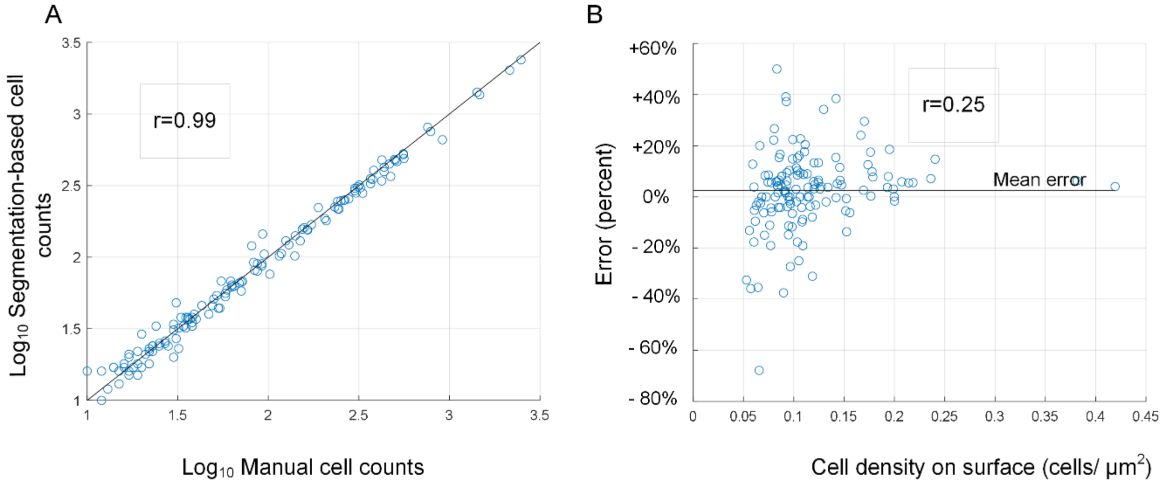

Figure S22. Aassessment of error bias in quantification of cell numbers in droplets. (A) Comparison of segmentation-based automated cell counts and manual cell counts. The strong correlation and alignment (r=0.99, Spearman correlation) along the diagonal indicate no systematic bias as a function of cell number. **(B)** Error (percent) as a function of cell density on the surface (cells/µm^2). No visible large bias is observed across the range of cell densities. Correlation is also quite low (r=0.25, Spearman correlation).

Supplementary Tables

Variable	Value	Comments
Total drop volume	$2.88*10^8$ μm^3	As in chip C6HD
Number of cells at t=0	15850 cells	As in chip C6HD
Droplet size distribution	Log normal	Similar to chip C6HD<br>Mean=$10^{4.15}$, Sigma =$10^{1.2}$
Population growth model	$\frac{dN}{dt} = r \cdot N \cdot \left(1 - \left(\frac{N}{K}\right)^m\right)$	Generalized logistic growth, with fitted parameters from the chip C6Hd:<br>r=0.55, m=0.61
Reproductive success relation	RS=a+b $\log_2$(Vol) + c $\log_2$(D0)	Based on the multiple regression of the experimental results of chip C6HD<br>a=-5.1<br>b= - 0.0357<br>c=-0.81;
Simulation time frame	0-24 hours	

Table. S1 Simulation settings and parameters in the simulation of chip C6HD as presented in the Results and Fig. 6.

Variable	Value	Comments
Total drop volume	10^9 µm^3	In the range of a representative chip
Number of cells at t=0 h	100,1000,10000, 100000	For each set of drop-size distribution simulated initial cell numbers spanned four orders of magnitude.
Droplet distributions (for Patchiness exploration)	Droplets were of equal volume	Droplet size values (in µm^3): 10^4,$10^{4.5}$,10^5,$10^{5.5}$,10^6,$10^{6.5}$,10^7,$10^{7.5}$,10^8, $10^{8.5}$,10^9
Droplet distribution ii (for Patch-size heterogeneity exploration)	Droplets followed log-normal distribution with mu=10^3.33 µm^3	Sigma values: 1.1, 1.2, 1.5, 2, 3, 5, 10, 20, 50, 100
Population growth model	$\frac{dN}{dt} = r \cdot N \cdot \left(1 - \left(\frac{N}{K}\right)^m\right)$	Generalized logistic growth, with fitted parameters from the chip C6HD: r=0.55, m=0.61
Reproductive success relation	RS=a+b $\log_2$(Vol) + c $\log_2$(D0)	As in chip C6HD experimental results a=-5.1 b= - 0.0357 c=-0.81;
Simulation time frame	0-48 hours	

Table S2. Simulation settings and parameters exploring the impact of patchiness and patch-size heterogeneity on population dynamics, as shown in Fig. 7.

**Chip**	**mu**	**sigma**
C1LD	3.75	1.064
C2LD	3.46	1.015
C3LD	3.25	1.045
C4LD	3.18	1.084
C5LD	3.28	1.095
C6HD	3.69	1.044
C7HD	3.2	1.035
C8HD	3.24	1.106
C9HD	3.18	1.025
C10HD	3.21	1.044
C11HD	3.20	1.037

Table S3. The estimated mu and sigma of the log-transformed droplet volume distribution for each of the 11 chips.

	Coefficient	Std error	t	P>|t|
**Constant (A)**	-5.0972	0.161	-31.700	0.000
**log2_Volume (B)**	-0.0357	0.010	-3.586	0.000
**log2_Density (C)**	-0.8108	0.018	-45.387	0.000

Table S4. Coefficients, standard errors, and t-statistic for reproductive success, droplet volume and initial density multiple regressions.

Chip		All droplet	Populated droplets	Initial cell no.	Final cell number	RS	Overall volume	Initial OD	Initial density	Initial Distribution	
										mu	sigma
A1_30 0623	C1 LD	902	182	4479	644844	7.17	3.94e+08	0.01	1.1e-5	3.75	1.231
B3_100 423	C2 LD	3051	507	6475	956225	7.21	5.19e+08	0.01	1.2e-5	3.46	1.124
C3_100 423	C3 LD	2215	331	7033	955052	7.09	4.78e+08	0.01	1.5e-5	3.25	1.111
A2_26 0623	C4 LD	1590	271	4382	505762	6.85	1.69e+08	0.01	2.6e-5	3.18	1.148
A2_25 0623	C5 LD	1139	225	8729	629987	6.17	2.24e+08	0.01	3.9e-5	3.28	1.169
B1_300 623	C6 HD	1111	404	15837	714708	5.5	2.88e+08	0.03	5.5e-5	3.69	1.153
B2_020 723	C7 HD	1498	412	12408	534297	5.43	1.44e+08	0.03	8.6e-5	3.2	1.098
B2_300 623	C8 HD	1101	312	20345	498677	4.62	1.9e+08	0.03	1.07e-4	3.24	1.179
B2_260 623	C9 HD	1777	474	18633	576445	4.95	1.69e+08	0.03	1.1e-4	3.18	1.084
B2_200 623	C10 HD	1229	307	37365	852088	4.51	2.4e+08	0.03	1.56e-4	3.21	1.114
B2_250 623	C11 HD	1180	409	34158	656372	4.26	1.54e+08	0.03	2.22e-4	3.20	1.103

Table S5. Cell numbers and droplet data from all 11 chips

Chip	Coefficient		Std error		t		P>|t|		R^2	
	Initial cell count	Carrying capacity	Initial cell count	Carrying capacity	Initial cell count	Carrying capacity	Initial cell count	Carrying capacity	Initial cell count	Carrying capacity
C1LD	0.89	0.88	0.04	0.01	19.98	74.18	1.87E-33	7.06E-137	0.83	0.97
C2LD	0.85	0.88	0.03	0.01	31.4	129.3	1.87E-82	0	0.82	0.97
C3LD	0.96	0.9	0.03	0.01	31.89	105.1	1.04E-57	3.35E-255	0.9	0.97
C4LD	0.92	0.91	0.05	0.01	18.1	97.32	1.77E-34	1.06E-211	0.75	0.97
C5LD	1	0.91	0.04	0.01	22.27	88.24	1.53E-35	2.10E-175	0.86	0.97
C6HD	0.96	0.88	0.03	0.01	30.17	115.8	8.85E-51	8.15e-311	0.9	0.97
C7HD	1.03	0.96	0.03	0.01	30.17	145.4	7.47E-48	0	0.91	0.98
C8HD	0.9	0.87	0.04	0.01	20.73	140.3	1.07E-33	1.68E-282	0.84	0.98
C9HD	0.97	0.92	0.02	0.01	39.56	127.5	5.71E-60	0	0.94	0.97
C10HD	1	0.94	0.03	0.01	31.95	145.5	6.08E-39	7.28E-284	0.95	0.99
C11HD	1.04	0.92	0.02	0.01	54.8	177.2	1.68E-55	0	0.98	0.99

Table S6. Summary of Ordinary Least Squares Regression analysis for initial and final cell counts across 11chips.

Chip	Log10 Vc	**Coefficient**		**Std error**		**t**		**P>|t|**		**R2**	
		Phase 1	Phase 2	Phase 1	Phase 2	Phase 1	Phase 2	Phase 1	Phase 2	Phase 1	Phase 2
C1LD	5.20	0.71	0.96	0.02	0.04	38.15	24.24	1.16E-69	2.25E-31	0.92	0.91
C2LD	5.30	0.71	0.96	0.01	0.02	60.34	44.55	1.52E-176	1.65E-100	0.92	0.92
C3LD	5.21	0.76	1.02	0.01	0.03	55.40	37.47	6.18E-141	6.30E-53	0.93	0.95
C4LD	5.12	0.78	1.05	0.02	0.03	44.37	36.63	1.63E-88	1.36E-65	0.93	0.92
C5LD	5..08	0.78	1.00	0.02	0.03	40.36	28.73	4.85E-80	3.08E-43	0.92	0.91
C6HD	4.81	0.74	0.96	0.01	0.02	52.77	48.19	3.75E-136	8.66E-95	0.92	0.94
C7HD	4.44	0.83	1.03	0.01	0.02	59.31	61.40	1.28E-154	1.26E-103	0.93	0.96
C8HD	4.80	0.78	0.92	0.02	0.01	49.20	73.21	1.99E-110	1.44E-99	0.93	0.98
C9HD	4.63	0.79	0.98	0.02	0.01	53.22	66.47	2.56E-161	1.19E-112	0.90	0.97
C10HD	4.61	0.85	0.96	0.01	0.02	62.43	50.97	2.53E-139	1.21E-67	0.95	0.97
C11HD	4.29	0.82	0.97	0.01	0.01	77.97	85.98	4.97E-203	2.82E-96	0.95	0.99

Table S7. Carrying capacity (*K*) as a function of droplet volume (*V*) show two phases of sub-linear relation.

Chip	Parameter	Coefficient	Std error	t	P>|t|
C1LD	Constant (A)	-4.778579	0.270509	-17.665155	4.43E-41
	log_2_Volume (B)	-0.021394	0.01427	-1.499262	1.36E-01
	log_2_Density (C)	-0.76594	0.025249	-30.334885	1.85E-72
C2LD	Constant (A)	-4.680549	0.153675	-30.457494	2.43E-116
	log_2_Volume (B)	0.010667	0.009459	1.127668	2.60E-01
	log_2_Density (C)	-0.718123	0.016798	-42.749833	9.32E-170
C3LD	Constant (A)	-4.855728	0.186442	-26.044124	7.67E-82
	log_2_Volume (B)	0.016657	0.010596	1.571993	1.17E-01
	log_2_Density (C)	-0.727482	0.019141	-38.005565	3.36E-122
C4LD	Constant (A)	-5.484374	0.186317	-29.435783	5.94E-86
	log_2_Volume (B)	0.009019	0.012812	0.703997	4.82E-01
	log_2_Density (C)	-0.795902	0.0207	-38.450074	4.42E-111
C5LD	Constant (A)	-5.038812	0.236391	-21.315591	1.29E-55
	log_2_Volume (B)	-0.00377	0.012328	-0.305815	7.60E-01
	log_2_Density (C)	-0.778164	0.023674	-32.870566	2.98E-87
C6HD	Constant (A)	-5.097174	0.160795	-31.699891	2.75E-111
	log_2_Volume (B)	-0.035683	0.009951	-3.585792	3.78E-04
	log_2_Density (C)	-0.81081	0.017864	-45.38714	4.48E-160
C7HD	Constant (A)	-5.944518	0.170932	-34.777005	3.13E-124
	log_2_Volume (B)	0.020344	0.007861	2.58797	1.00E-02
	log_2_Density (C)	-0.816115	0.017564	-46.465744	2.93E-165
C8HD	Constant (A)	-4.856769	0.139581	-34.79534	6.64E-109
	log_2_Volume (B)	-0.071935	0.00721	-9.97688	1.67E-20
	log_2_Density (C)	-0.828011	0.015467	-53.534418	2.29E-158

C9HD	Constant (A)	-5.162353	0.162532	-31.76205	3.63E-119
	log_2_Volume (B)	-0.003007	0.008982	-0.334716	7.38E-01
	log_2_Density (C)	-0.784406	0.01819	-43.122984	1.18E-165
C10HD	Constant (A)	-5.59527	0.167821	-33.34067	1.47E-103
	log_2_Volume (B)	-0.013102	0.007365	-1.779047	7.62E-02
	log_2_Density (C)	-0.837393	0.01784	-46.93954	2.54E-141
C11HD	Constant (A)	-5.33235	0.11138	-47.875095	4.58E-169
	log_2_Volume (B)	-0.035112	0.005512	-6.370473	5.11E-10
	log_2_Density (C)	-0.858319	0.011852	-72.417662	2.98E-234

Table S8. Reproductive Success (RS) Multiple Linear Regression coefficients across 11 chips.
